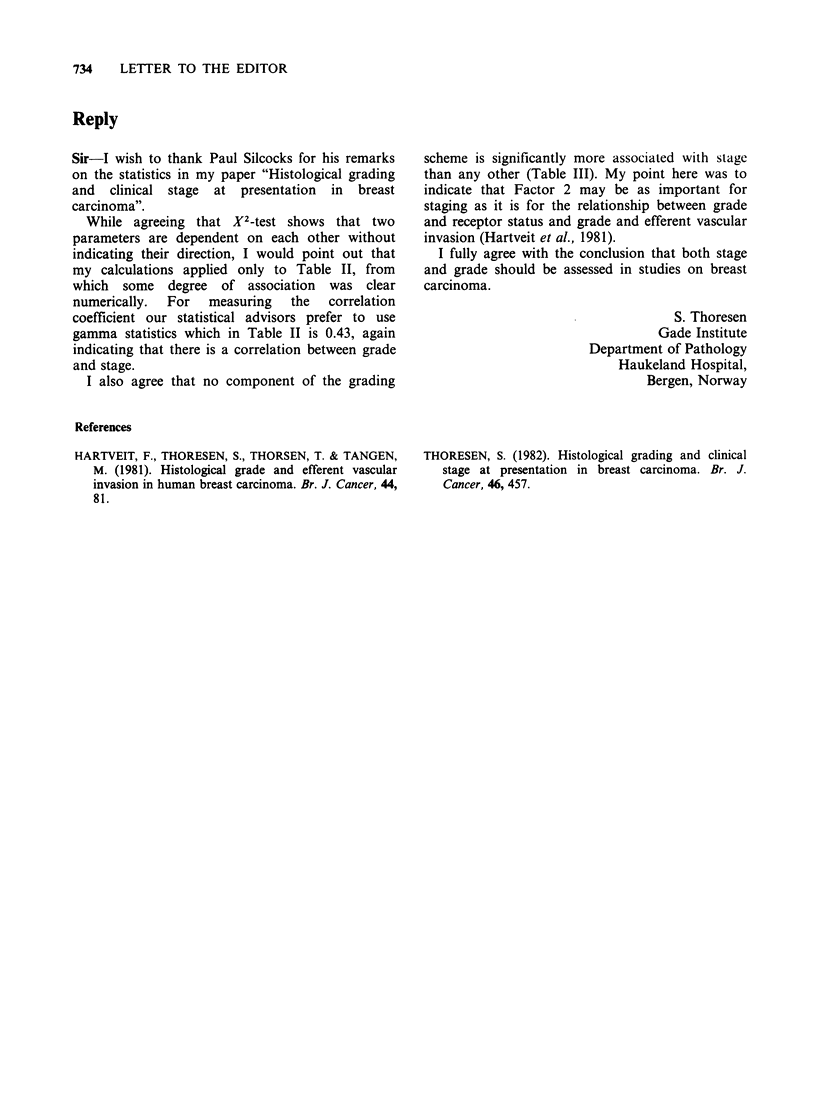# Reply

**Published:** 1983-05

**Authors:** S. Thoresen


### Reply

**Sir**—I wish to thank Paul Silcocks for his remarks on the statistics in my paper "Histological grading and clinical stage at presentation in breast carcinoma".

While agreeing that $X^2$-test shows that two parameters are dependent on each other without indicating their direction, I would point out that my calculations applied only to Table II, from which some degree of association was clear numerically. For measuring the correlation coefficient our statistical advisors prefer to use gamma statistics which in Table II is 0.43, again indicating that there is a correlation between grade and stage.

I also agree that no component of the grading scheme is significantly more associated with stage than any other (Table III). My point here was to indicate that Factor 2 may be as important for staging as it is for the relationship between grade and receptor status and grade and efferent vascular invasion (Hartveit *et al.*, 1981).

I fully agree with the conclusion that both stage and grade should be assessed in studies on breast carcinoma.

S. Thoresen
Gade Institute
Department of Pathology
Haukeland Hospital,
Bergen, Norway

**References**

HARTVEIT, F., THORESEN, S., THORSEN, T. & TANGEN, M. (1981). Histological grade and efferent vascular invasion in human breast carcinoma. *Br. J. Cancer*, **44**, 81.

THORESEN, S. (1982). Histological grading and clinical stage at presentation in breast carcinoma. *Br. J. Cancer*, **46**, 457.